# Adult prognosis of childhood kidney replacement therapy: ERA Registry Figure of the Month

**DOI:** 10.1093/ckj/sfaf204

**Published:** 2025-07-07

**Authors:** Vianda S Stel, Alberto Ortiz, Anneke Kramer

**Affiliations:** ERA Registry, Department of Medical Informatics, Amsterdam UMC—Location, University of Amsterdam, Amsterdam, the Netherlands; Amsterdam Public Health Research Institute, Quality of Care, Amsterdam, the Netherlands; Department of Nephrology and Hypertension, IIS-Fundacion Jimenez Diaz UAM, Madrid, Spain; Department of Medicine, Universidad Autonoma de Madrid, Madrid, Spain; ERA Registry, Department of Medical Informatics, Amsterdam UMC—Location, University of Amsterdam, Amsterdam, the Netherlands; Amsterdam Public Health Research Institute, Quality of Care, Amsterdam, the Netherlands

**Figure 1:**
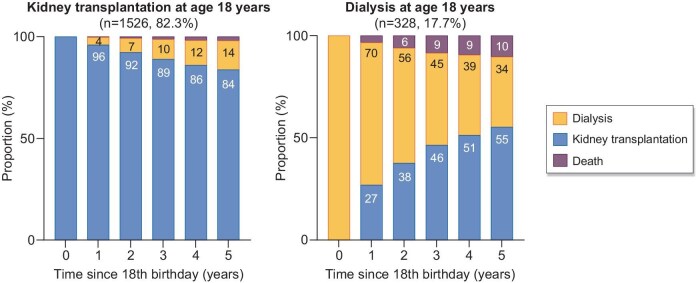
Prognosis of adult survivors of childhood KRT during the first 5 years after reaching age 18 years between 2008 and 2015 in Europe, by treatment. **Source:** Montez de Sousa et al. NDT 2025, https://doi.org/10.1093/ndt/gfae189, Fig. 3. The figure was slightly adapted from the original figure (only showing bars by year). **Explanation:** Most adult survivors of childhood kidney replacement therapy reached their 18th birthday with a functioning kidney transplant (82.3%). Of these, 14% switched to dialysis and 2% died within 5 years (prior to their 23rd birthday). Among patients on dialysis at their 18th birthday (17.7%), 55% received a kidney transplant, 34% remained on dialysis and 10% died within 5 years.

